# Riboflavin Ameliorates Cisplatin Induced Toxicities under Photoillumination

**DOI:** 10.1371/journal.pone.0036273

**Published:** 2012-05-02

**Authors:** Iftekhar Hassan, Sandesh Chibber, Aijaz A. Khan, Imrana Naseem

**Affiliations:** 1 Department of Biochemistry, Faculty of Life Sciences, Aligarh Muslim University, Aligarh, India; 2 Department of Anatomy, Jawaharlal Nehru Medical College, Aligarh Muslim University, Aligarh, India; National Institutes of Health, United States of America

## Abstract

**Background:**

Cisplatin is an effective anticancer drug that elicits many side effects mainly due to induction of oxidative and nitrosative stresses during prolonged chemotherapy. The severity of these side effects consequently restricts its clinical use under long term treatment. Riboflavin is an essential vitamin used in various metabolic redox reactions in the form of flavin adenine dinucleotide and flavin mononucleotide. Besides, it has excellent photosensitizing property that can be used to ameliorate these toxicities in mice under photodynamic therapy.

**Methods and Findings:**

Riboflavin, cisplatin and their combinations were given to the separate groups of mice under photoilluminated condition under specific treatment regime. Their kidney and liver were excised for comet assay and histopathological studies. Furthermore, Fourier Transform Infrared Spectroscopy of riboflavin-cisplatin combination *in vitro* was also conducted to investigate any possible interaction between the two compounds. Their comet assay and histopathological examination revealed that riboflavin in combination with cisplatin was able to protect the tissues from cisplatin induced toxicities and damages. Moreover, Fourier Transform Infrared Spectroscopy analysis of the combination indicated a strong molecular interaction among their constituent groups that may be assigned for the protective effect of the combination in the treated animals.

**Conclusion:**

Inclusion of riboflavin diminishes cisplatin induced toxicities which may possibly make the cisplatin-riboflavin combination, an effective treatment strategy under chemoradiotherapy in pronouncing its antineoplastic activity and sensitivity towards the cancer cells as compared to cisplatin alone.

## Introduction

Cisplatin (CP) or cis- diamminedichloroplatinum (II), a platinum based antineoplastic agent, is one of the prominent members of the most effective broad spectrum anticancer drugs used against cervical, head and neck, prostrate, breast, lung, testicular and ovarian cancers [1]. The anticancer activity of this drug is attributed to its capability to form covalent bonds at N-7 position of purine residues of DNA leading to formation of 1, 2 or 1, 3-intrastrand crosslinks and a lesser extent of the interstrand crosslinks. These adducts of CP-DNA derail the cellular replication and transcription machinery if these lesions anyhow evade DNA repair system in the effected cells [2]. Many labs including ours have demonstrated that CP generates free radicals leading to oxidative and nitrosative stress which results into such deleterious effects *in vivo* [3], [4].

Riboflavin (RF) or vitamin B_2_ is an essential vitamin that is required for normal cellular functions, growth and development in all aerobic forms of life. It occurs in two major forms- flavin adenine mononucleotide (FMN) and flavin adenine dinucleotide (FAD) that participate in various metabolic redox reactions including electron transport chain in mitochondria and also as a prosthetic group of many enzymes like glutathione reductase and succinate dehydrogenase. It can undergo photolysis and photoaddition leading to generation of various free radicals having potential to exert derogatory effects on the macromolecules *in vitro* and *in vivo* [5], [6], [7]. Furthermore, being an excellent photosensitizer, it is used in photodynamic therapy (PDT) and ribophototherapy (RPT) for treatment of various diseases including cancer [8], [9]. Interestingly, deficiency of this vitamin has been ascribed to play a prominent role in progression of various cancers as well as increased vulnerability of the cells to cancer [10]. RF has been manifested to enhance antitumor activity of many anticancer drugs as well as in boosting the immune system to kill tumor cells [11], [12].

Despite being an effective antiproliferative agent, the clinical usage of CP is limited by various side effects including nephrotoxicity, hepatotoxicity, neurotoxicity and ototoxicity. They force the patients either to limit its dose or discontinue its use during long term CP based chemotherapy. Hence, amelioration or decreasing the severity of CP elicited toxicity is one the major clinical challenges in the cancer research arena for the last two decades and many strategies have been tried ever since [1], [13], [14], [15]. In the recent past, our lab has demonstrated that the photosensitizing property of RF can be used to alleviate the toxicities induced by CP *in situ* and *in vivo* [4], [16]. Our present work is the first to study the blunting effect of cisplatin-riboflavin combination on cisplatin induced toxicities *in vivo* at the molecular level.

## Materials and Methods

### Materials

Cisplatin, riboflavin, normal melting agarose (NMA), ethidium bromide (EtBr), Histopaque 1077, Hank's balanced salt (HBSS), RPMI 1640 and low melting point agarose (LMPA) were purchased from Sigma- Aldrich Chemical Company, USA. Ethylenediaminetetraacetic acid (EDTA), triton X-100, tris- HCl, NaCl, Na_2_HPO_4_, NaH_2_PO_4_ and NaOH were bought from Qualigens Fine chemical company, India. All other chemicals used were purchased from Sisco Research Lab, Mumbai and HiMedia Laboratories Private Limited, Mumbai.

### Animal husbandry and treatment

Forty two adult Swiss albino male mice of 6 months' age weighing 48–50 g were purchased from the Central Animal House of Jamia Hamdard University, New Delhi, India. . The animals were housed in sufficiently large cages and treated under humane and hygienic conditions with maintained 25±2°C and 12 hours day: night cycle according to ‘Departmental Ethical Committee for Animal Experimentation’. They were acclimatized for 10 days before the treatment on standard pellet mice diet (Ashirwad Industries, Chandigarh, India) and clean drinking water *ad libitum*.

Animal experimentations were permitted by Ministry of Environment and Forests, Government of India under registration no 714/02/a/CPCSEA issued by Committee for the Purpose of Control and Supervision of Experiments on Animals (CPCSEA) dated 16th November, 2002. All experiments on animals were approved by Departmental Ethical Committee (acad/D-833/ILK/07-09-2007).

They were divided into 7 groups randomly taking 6 mice in each group. They were named as- group I [control], group II [treated with riboflavin at the dose of 2 mg/kg body weight], group III [treated with cisplatin at the dose of 2 mg/kg body weight]. The combination of CP and RF was named as group IV [treated with the dose of 2 mg/kg body weight of CP with 1 mg/kg body weight of RF (CP+RF1)] and Group V [treated with the dose of 2 mg/kg body weight of CP with 2 mg/kg body weight of RF (CP+RF2)] respectively. Group I was injected with saline only in equal volume of the dose given to the treatment groups. All these groups were exposed to full body irradiation under florescent light [Philips, India] kept at ∼10 cm distance at fluence rate of 38.6 W/m^2^ for 12 hours daily during daytime. Parallel to these, additional two combination groups without exposure of light - IV′ (CP+RF1) and V′ (CP+RF2) were also maintained. All the doses were injected intraperitonially with 1 ml capacity syringe using saline as vehicle solution for all the treatment. RF was injected prior to CP by ½ hour in all the combination treated groups [IV, IV′, V and V′]. The dorsal surface of all the mice was mildly shaved for maximum possible absorption of light through the skin. The mice were given a daily injection for 3 days followed by a gap of a week; then again a daily dose for 3 days with a week gap and finally 3 more daily injections were given. This treatment schedule was applied to mimic the current cancer treatment strategy for the patients. The treatment strategy, dose and the duration of treatment were chosen to study the chronic effect of the treatment at moderately toxic dose of the drug. Group I was taken as the control for comparison with all other groups. All the mice were healthy during the whole treatment. All the animals were sacrificed on the same day by cervical dislocation method on the next day to the final dose given.

### Preparation of samples

After the sacrifice, their kidneys and livers were washed with ice-cold saline buffer. Each of the samples was cut with sterilized blade into two parts: one portion was kept in HBSS for comet assay while the rest was stored in 10% formalin for their histopathological studies.

For comet assay, the organs were submerged in HBSS with a pinch of EDTA and RPMI 1640 followed by their chopping into small cubes of 2–3 nm thickness in separate Petri dishes. Their solutions were sieved by muslin cloth into fresh Petri dishes to collect their cell suspension and were properly labeled. Cell viability test was conducted for all the samples by trypan blue exclusion method.

### Procedure of comet Assay

The assay was performed in alkaline condition in accordance with protocol of Singh et al. with few modifications. Fully frosted slides precoated with 1% NMA (as base layer) at 60°C were prepared a day before sacrificing the animals. About 10,000 cells isolated from each organ cell suspension were mixed with 100 µl 1% of LMPA to form the working cell suspension separately for each organ. This suspension was pipetted over the base layer at 37°C followed by covering with cover slips immediately. After solidification of second layer by keeping on ice packs, the cover slips were removed and a third layer of 0.5% LMPA (100 µl) was pipetted over followed by covering with cover slips and kept on ice packs again. After that, the cover slips were removed and the slides were immerged in cold lysing solution (2.5 M NaCl+100 mM EDTA+10 mM tris-base+1% triton X-100) of pH 10 and were kept as such for 3 hours. They were allowed to unwind in alkaline electrophoretic running buffer (300 mM NaOH + 1 mM EDTA) having pH 13 in electrophoretic tank for 30 minutes. Then, electrophoresis was performed for 35 minutes at 4°C with constant field strength of 0.74 volts/cm and current strength of 300 mA. After gently washing with cold saline thrice, the slides were placed in neutralizing buffer (0.4 M tris-base) of pH 7.5 followed by washing with cold saline. The process of neutralization followed by washing was repeated thrice. Their staining was done with 80 µl ethidium bromide (20 µg/ml) for 5 minutes. Finally, the slides were washed with chilled saline for three times and cover slips were placed on. They were kept in humidified slide box in refrigerator and were analyzed on the next day. The slides were scored with CX41 fluorescent microscope (Olympus, Japan) coupled with an image analysis system (Komet 5.5, Kinetic imaging, Liverpool, U.K.) that was attached to integrated CC camera COHU 4910 (equipped with 510–560 nm excitation and 590 nm barrier filters). The comets were scored at the magnification of 100× and images of 50 cells (25 from each replicate slide) for each sample were scored. Comet tail-length (migration of DNA from its nucleus in µm) was chosen as the parameter to assess the nuclear DNA damage for the present study.

### Histopathological study of kidney and liver samples of treated mice

For histopathology, the kidney and liver samples were kept in 10% formalin for immersion fixation. 10×5×3 mm sized tissue blocks of both the organs were processed for paraffin embedding. Their sections of 7 micrometer (µm) thickness were cut with rotary microtome and stained with Hematoxylin and Eosin stain. The sections were observed under trinocular light microscope (Olympus BX40, Japan) and their photomicrographs were snapped at magnification of ×400. After sectioning of the organs, five randomly selected sections from each group of both organs were subjected to counting of cells under light microscopy manually. The cells showing normal shape and size with clear and well defined nucleus and intact cytoplasm were considered as normal cells. The cells demonstrating shrinkage or partial/complete disappearance of nucleus were considered as apoptotic cells whereas cells showing fuzzy nucleus and scanty cytoplasm were assumed as cells undergoing necrosis.

### Fourier Transform Infrared Spectroscopy (FT-IR) of RF, CP and their combinations

To get the insight picture of any possible interaction between the two compounds, the aqueous solution of RF (50 µM), CP (50 µM) and their combination were prepared freshly followed by their incubation under fluorescent light for 1 hour parallel with their controls without any light exposure. The samples were sandwiched between two potassium bromide discs by hydraulic pressing and their infrared spectra were recorded by Shimadzu-8300 FTIR spectrophotometer (Tokyo, Japan). Their scanning range was set at 600–2000 cm^−1^ with resolution of 4 cm^−1^.

### Statistical analysis

All the data has been expressed in mean ± SEM (standard error of mean). Comparisons among various groups were conducted by one way –ANOVA with the help of software ‘Origin 6.1’ and ‘GraphPad Prism 5’. p ≤0.05 was chosen as statistically significant for the treatment. The experiments were repeated thrice to check the reproducibility of the results.

## Results

### CP- RF combination demonstrated better recovery from CP induced nuclear DNA damage in kidney and liver samples as assessed by comet assay

**Figure 1:**
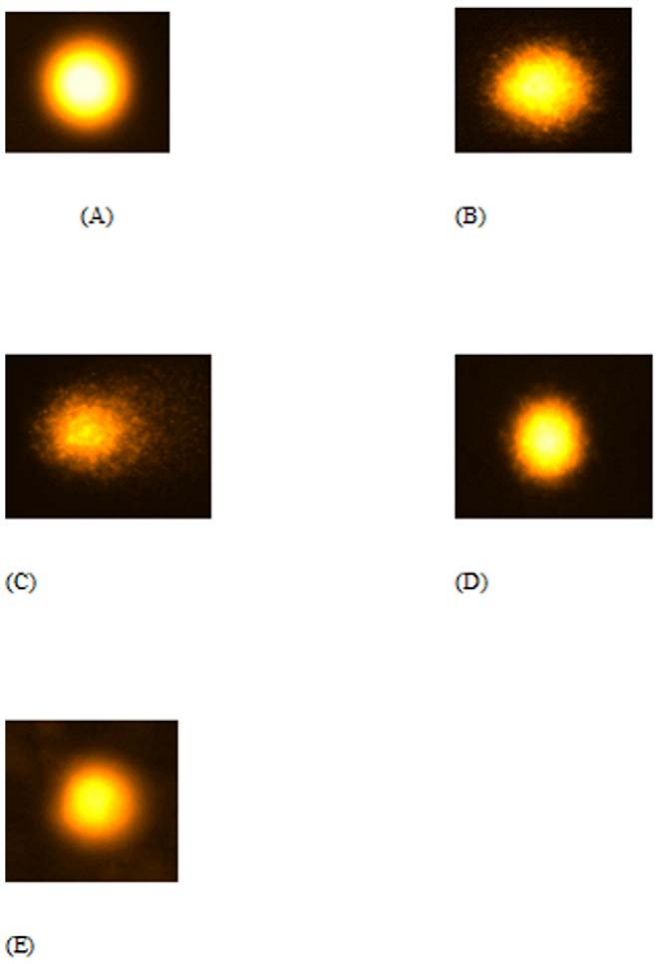
Showing the average comet picture of kidney from different treated groups of mice. Average comet picture of kidney samples of various treated mice groups divided as (A) Control as Group I (B) Riboflavin treated as Group II (C) Cisplatin treated as Group III (D) Combination I as Group IV (E) Combination II as Group V.

**Figure 2:**
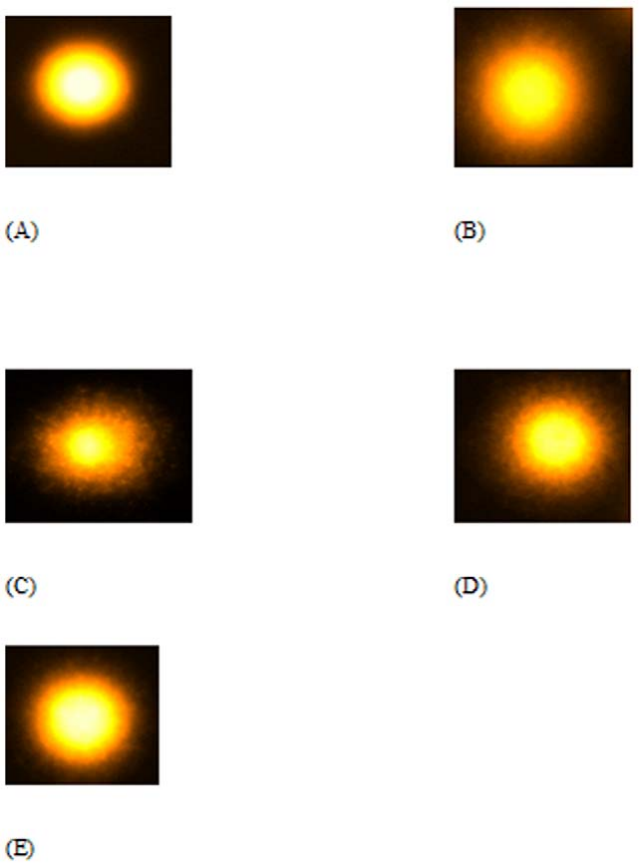
Showing the average comet picture of liver from different treated groups of mice. Average comet picture of liver samples of various treated mice groups divided as (A) Control as Group I (B) Riboflavin treated as Group II (C) Cisplatin treated as Group III (D) Combination I as Group IV (E) Combination II as Group V.

CP and RF treatment under photoillumination caused major DNA damage in the kidney and liver samples as evidenced by elongated tail length in group III and II with respect to the control (group I). Group II showed increase in tail length in kidney and liver cells by 115.8% and 103% [Figure 1(B) and 2(B)] while group III demonstrated the increment in the tail length in kidney and liver cells by 231.6% and 218.2% respectively [Figure 1(C) and 2(C)]. The combination groups under photoillumination showed dose dependent recovery in CP induced DNA damage as group IV and V demonstrated decrease in the tail length by 26.6% and 55.6% in the kidney cells [Figure 1(D) and 1(E)] while 28.6% and 59.5% in the liver cells respectively [Figure 2(D) and 2(E)] as compared to group III. Among the combination groups without light exposure, group V′ displayed 13.5% and 16.2% recovery in tail-length in kidney and liver cells followed by group IV′ showing the recovery by 6.4% and 7.6% in kidney and liver cells respectively (Figures not shown) indicating the better recovery under the effect of light (Table 1; Table S1).

**Table 1:** Showing the average tail length of comet of the treated cells of different groups.

Name of Group	Treatment	Average tail-length of kidney cells (in µm)	Average tail-length of liver cells (in µm)
I	Saline	7.6±1.65	6.6±1.7
II	Riboflavin (2 mg/kg)	16.4**±1	13.4*±1.5
III	Cisplatin (2 mg/kg)	25.2**±2. 5	21.0**±1.2
IV	CP(2 mg/kg)+RF(1 mg/kg)	18.5##±1.2	15.0#±1.5
IV′	CP(2 mg/kg)+RF(1 mg/kg)	23.6#±1.4	19.4#±2
V	CP(2 mg/kg)+RF(2 mg/kg)	11.2##±1.5	8.5##±1. 5
V′	CP(2 mg/kg)+RF(2 mg/kg)	21.8#±1.2	17.6#±2

All the data have been expressed in mean ±SEM for six different preparation of each sample of three independent experiments.

* indicates significantly different from control at p≤0.05.

** indicates significantly different from control at p≤0.01.

# indicates significantly different from group III at p≤0.05.

## indicates significantly different from group III at p≤0.01.

### Histopathological studies showed healing effect of cisplatin induced tissue damage by riboflavin in the combination treated group

**Figure 3:**
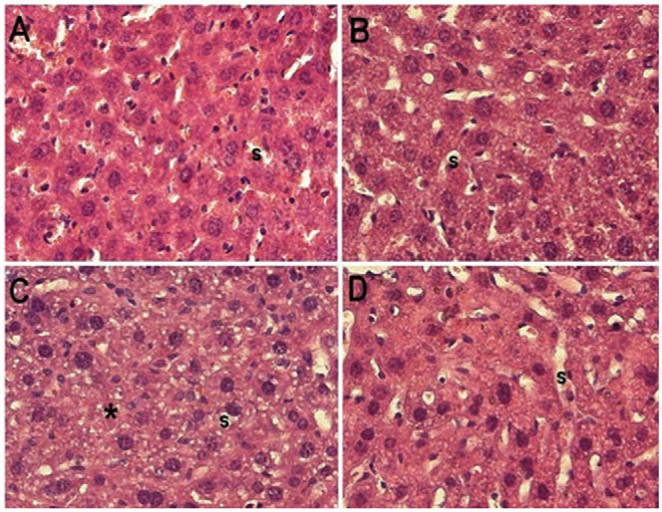
Histomicrographs of mice liver of major groups. Showing histomicrographs of liver samples of various groups indicated in parentheses. All the sections have been stained with Hematoxylin and Eosin stain and were snapped at 400×. (A) Control [group I] depicts normal hepatocellular structure and sinusoids (s). (B) Riboflavin treated [group II] shows features very akin to normal. (C) Cisplatin treated [group III] reveals microvesicular changes in the hepatocytes as well as regions devoid of hepatocytes (*). (D) The combination of cisplatin and riboflavin treated [group IV] again shows features quite similar to normal control.

The liver histomicrograph of group II [Figure 3(B)] was appeared to be normal having the contour of hepatocytes intact while sinusoids remained patent without any sign of congestion except enlargement of the hepatocytes with respect to the control [Figure 3(A)]. The liver section of CP treated group III showed prominent alteration in the hepatic microstructure including altered contour of hepatocytes, sparse presence of cell organelles, shrunken nuclei, collapsed sinusoids and poorly maintained hepatic cords [Figure 3(C)]. These cellular features indicate that the cells were severely damaged and most of them were either undergoing apoptotic or necrotic pathway. Group IV liver histomicrograph revealed intact hepatic cords, hepatocyte contour and patent sinusoids [Figure 3(D)] which was closely comparable to the control group. These are suggestive of ameliorating effect of riboflavin in CP-RF combination treated mice under photoilluminated condition [Figure 3(D)] while its counterpart; the combination treated group without photoillumination didn't show any significant improvement in their tissue sections [Figures not shown].

**Figure 4:**
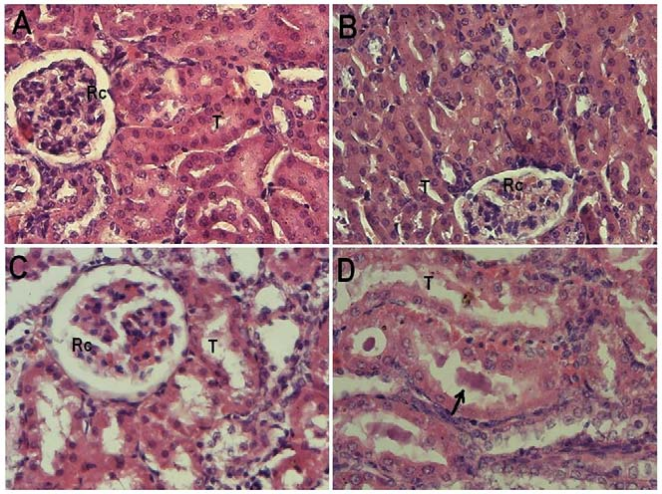
Histomicrograph of mice kidney of major groups. Showing histomicrographs of kidney samples of various groups indicated in parentheses. All the sections have been stained with Hematoxylin and Eosin stain and were snapped at 400×. (A) Control [group I] depicts normal renal corpuscles (Rc) and tubules (T). (B) Riboflavin treated [group II] shows features very akin to normal. (C) Cisplatin treated [group III] reveals changes suggestive of dilatation of tubules, and nuclear cytoplasmic dissociation in the tubular epithelium. (D) The combination of cisplatin and riboflavin treated [group IV] shows reasonably intact tubular epithelium and lumen filled with cast (↑).

The kidney section from group II [Figure 4(B)] was found very much comparable to the control group [Figure 4(A)] showing no any evident change in its microanatomy of renal tubules and renal corpuscles. Kidney histomicrograph of the CP treated group III showed structural features of acute tubular necrosis characterized by swelling which could be due to loss of apical portion or separation tubular epithelium from its basement membrane at various sites [Figure 4(C)]. The combination treated group IV histomicrograph depicted renal tubules as normal comparable the control. However, the lumina of some of their tubules showed renal casts possibly arising from the sloughing of tubular epithelium. In addition, the lining of epithelial cells appeared flat as compared to the control. Hence, the combination treated group IV exhibited less cisplatin induced structural aberrations which is indicative of the ameliorative effect of riboflavin in the group [Figure 4(D)].

After qualitative histopathological examination of the tissue sections, semiquantitative cell counting was performed on randomly selected five tissue sections of both the kidney and liver from all the groups. As kidney parenchyma consists of diverse type of cells; the cells per renal tubule epithelium were taken as the parameter to count the cells. It was observed that number of cells showing necrotic features was highest in group III followed by group II while the cells showing apoptotic features was more prominent in group IV with decrease in number of cells undergoing necrosis as compared to group III.

**Figure 5:**
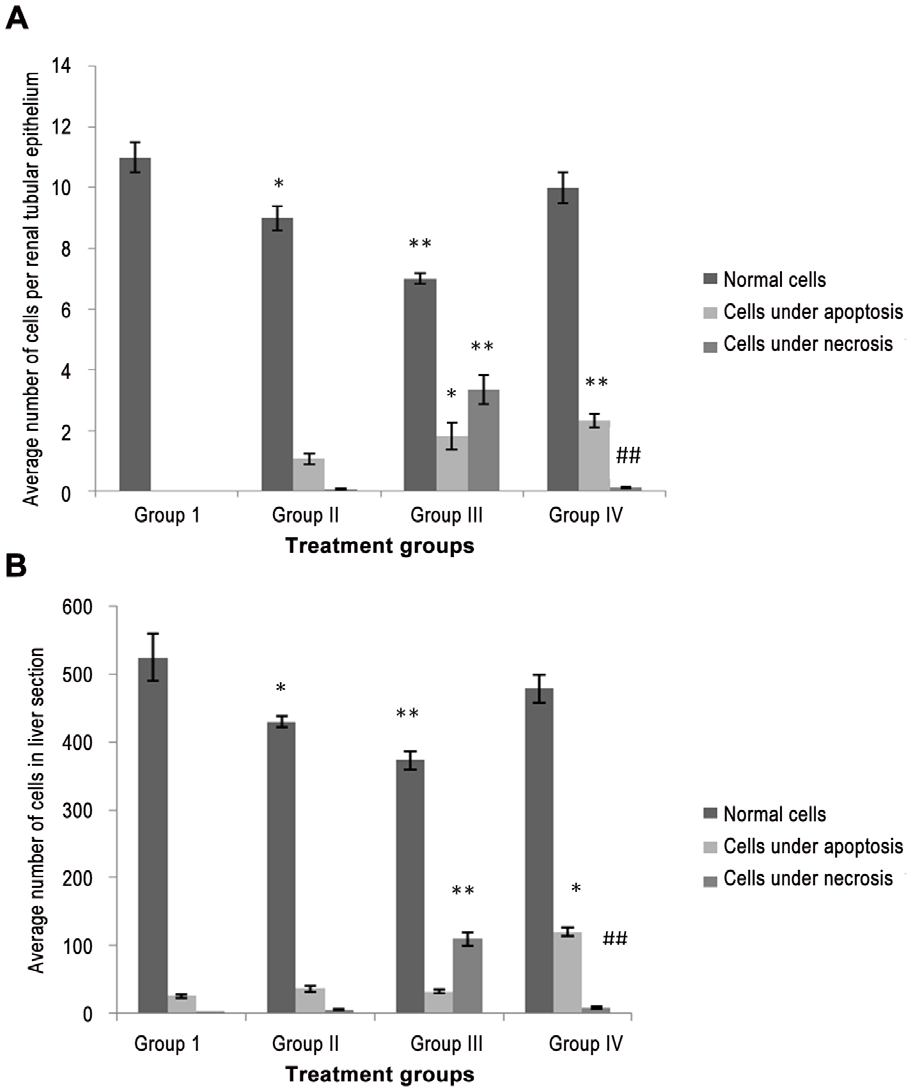
**(A): Bar diagram showing average number of cells per renal tubular epithelial cell in kidney section of various groups.** All the data have been expressed in mean ±SEM for five different preparation of each sample. * indicates significantly different from control at p≤0.05. ** indicates significantly different from control at p≤0.01. # indicates significantly different from group III at p≤0.05. **##** indicates significantly different from group III at p≤0.01. **(B). Bar diagram showing average number of cells in liver section of various groups.** All the data have been expressed in mean ±SEM for five different preparation of each sample. * indicates significantly different from control at p≤0.05. ** indicates significantly different from control at p≤0.01. # indicates significantly different from group III at p≤0.05. **##** indicates significantly different from group III at p≤0.01.

In liver, because of the uniformity in the cellular arrangement all hepatocytes in a section were taken into account for their counting. The number of cells under necrosis was highest in group III while group IV demonstrated the highest number of cells under apoptosis, a pattern very similar to one observed in kidney sections [Figure 5(A) and 5(B); Figure S1].

### FTIR results and analysis

FTIR of combination -II showed that there is a strong interaction between CP and RF in both the cases. RF showed a characteristic peak of –NH_2_ (amide III) group of the alloxazine ring at 1240.02 cm^−1^ while CP displayed its characteristic peaks at and 1558.93 and 1539.24 cm^−1^ for both of its –NH_2_ (amide II) group [17], [18]. These peaks demonstrated noticeable shift in case of their combinations under photoilluminated condition. Hitherto, the combination without light exposure didn't show any significant shift in the peaks. It indicates involvement of the interaction between the active groups of both the compounds at the molecular level was pronounced in presence of light.

**Figure 6:**
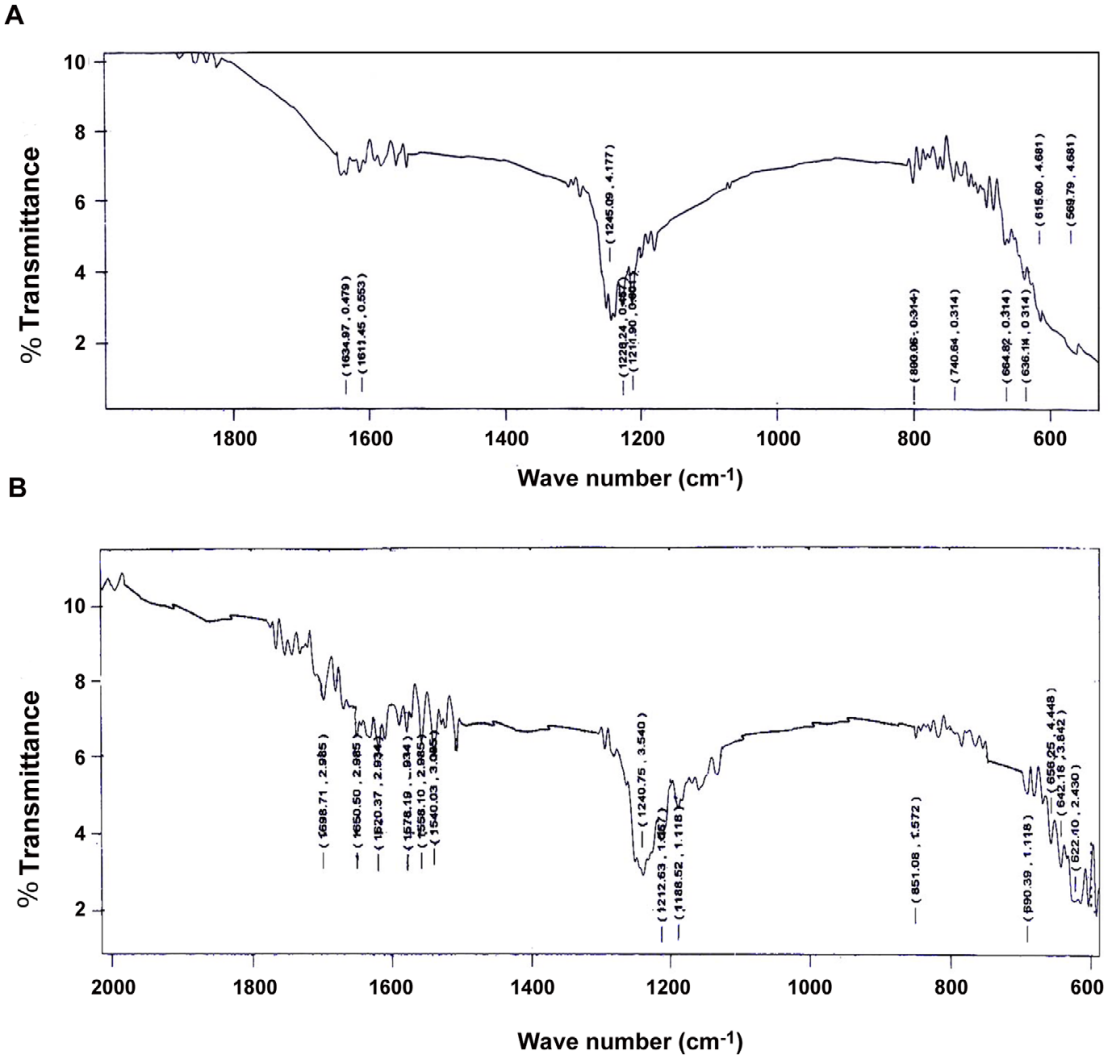
FTIR of CP-RF combination with and without photoillumination. Showing infrared spectra of CP-RF combination in presence and absence of photoillumination taking wave number (cm^−1^) and % transmission (%T) at X and Y-axes respectively. (A) FTIR spectra of CP-RF combination without incubation under light. (B) FTIR spectra of CP-RF combination after I hour of incubation under light.

The combination of CP and RF without photoillumination showed poor interaction between two compounds as mild alteration was observed in their characteristic peaks of all the active groups [at 1648.49 and 1630.41 cm^−1^ (for C = O group) and 1240.02 cm^−1^ (for –NH group) of alloxazine ring of RF; 1245.85, 1234.96, 1539.24 and 1558.93 cm^−1^ for two -NH_2_ groups while 676.81 and 720.12 cm^−1^ for two chloride groups of CP] (Figures not shown). Hitherto, the combination generated a series of new peaks (from 1698.71 to 1540.03 cm^−1^ for –NH groups and 1212.63 and 1188.52 cm^−1^ for C = O groups) after 1 hour of light exposure [17], [18]. The generation of these novel peaks indicates that an interaction existed between RF and CP that increased by many folds after photoillumination [Fig. 6(A) and 6(B)].

## Discussion

During prolonged chemotherapy, the clinical usage of CP is restricted or discontinued because of its severe side effects like nephrotoxicity, hepatotoxicity, neurotoxicity and ototoxicity [2], [4], [19], [20]. Their amelioration has been a great challenge for medical research since its clinical implementation. The present investigation is aimed to make CP based chemoradiotherapy more effective and less toxic in the same league [19], [20]. RF is an important ubiquitously occurring vitamin in all aerobes. Recent studies have demonstrated that RF is a potential apoptosis inducer in various cancerous strains and have been recommended as an effective adjuvant therapeutic agent in the treatment of diverse class of diseases including cancers [21]–[24].

Our present work for the first time demonstrates that CP-RF combination is significantly less toxic as demonstrated by comet assay and histopathology of liver and kidney samples of treated animals. These results are also supported by our previous study in which all the major oxidative stress parameters and target organ functional markers were almost normalized when RF was used with CP *in vivo* [7] which is also in agreement with our previous investigation on mice keratinocytes [16].

Cancer cell lines based studies conducted by various investigators have demonstrated that riboflavin can activate extrinsic apoptosis pathway at low concentration in their culture but additional cell death mechanisms like intrinsic apoptotic pathway and ubiquitin/proteasome pathway are also triggered at higher concentration of RF leading to further inhibition in their proliferation [20], [21], [25]. RF has also been implicated in downregulation of many anti-apoptotic factors like P13K, JNK and ERK ½ phosphorylation as well as upregulation of many other apoptosis inducing factors like cytochrome *C*, Smac/Diablo and htr A2/Omi. Recently, RF has also been shown to promote extensive vacuole formation in HeLa and HL-60 cells indicating involvement of autophagy parallel to intrinsic and extrinsic pathways of apoptosis [26]. Hence, it is expected that all modes of cell death might get involved when RF was present in the cancer cell culture. This potential of RF can add teeth to anticancer drug like CP when used to combat the cancer cells *in vivo* as well.

The CP toxicity in target organs including kidney and liver is very complex and involves network of various interrelated mechanisms. CP is supposed to bind with cellular reductants and proteins inside the cells leading to accumulation of ROS. The invasion of ROS on the cells causes inflammation locally via upregulation of pro-inflammatory cytokines (TNF-α, IL-1β and IL-6) as well as chemokines (CXCL1/KC) that triggers expression of iNOS as well as the adhesion molecules (e.g., ICAM-1) in the target cells [27]. It leads to NO generation and adhesion molecules facilitate the recruitment of inflammatory cells for inflammatory response in the target cells. Besides, CP mediated inflammation also enhance the expression of ROS generating phagocytic NADPH oxidases like NOX4 and NOX2 [28], [29]. Hence, oxidative and nitrosative stress both play roles in CP-mediated toxic insults. Furthermore, these stresses can activate interrelated downstream pathways such as the nuclear enzyme PARP that is considered as a hallmark of irreversible apoptosis induction [30]. As literature suggests that higher oxidative and nitrosative stress triggers necrosis while its low to moderate level induces programmed cell death [31], it is possible in our case that riboflavin is either directly decreasing the stresses by replenishment of cellular reducing powers and antioxidant enzymes or maybe it is not allowing CP to cause any organ injury thereby avoiding any derogatory inflammatory response.

CP also binds to DNA leading to its damage triggering p53 phosphorhylation and activation. After activation, the p53 mediated downstream regulation mechanism can be divided in two categories: transcription-independent (mitochondrial and cytosolic) and transcription dependent (nuclear). By transcriptional regulation, nuclear p53 can activate proapoptotic genes like PUMA-α, PIDD, caspases and ER-iPLA as well as can also repress antiapoptotic genes like p21 and taurine transporter (Tau T). Besides, it is also speculated that p53 may also induce cell death by transcription independent mechanisms via interaction with Bcl-2 family proteins in mitochondria or cytosol [32]. Moreover, various studies entail that increased oxidative and nitrosative stresses can directly trigger nuclear p53 gene to avoid any DNA damage or enter apoptosis depending upon the severity of damage and cell types [31]. However, it is proved that excessive free radicals can perturb mitochondria and can induce cytochrome C -mediated apoptosis. In our case, it is possible that RF or CP-RF combination can favor the apoptosis inducing pathways or it may bring down the stress level to threshold level which favors apoptosis instead of necrosis. The results with combination treated mice indicate this possibility in the present investigation. Furthermore, reduction in necrotic cell death in group IV organ samples indicates that tissue damage is minimized with RF supplementation. In addition, the increase in apoptosis could be index towards the increase in malignant cell death providing explanation to our preliminary data on BC-3 cell line in which CP-RF combination has shown plausible enhancement in cell death under MTT assay (data not published).

**Figure 7:**
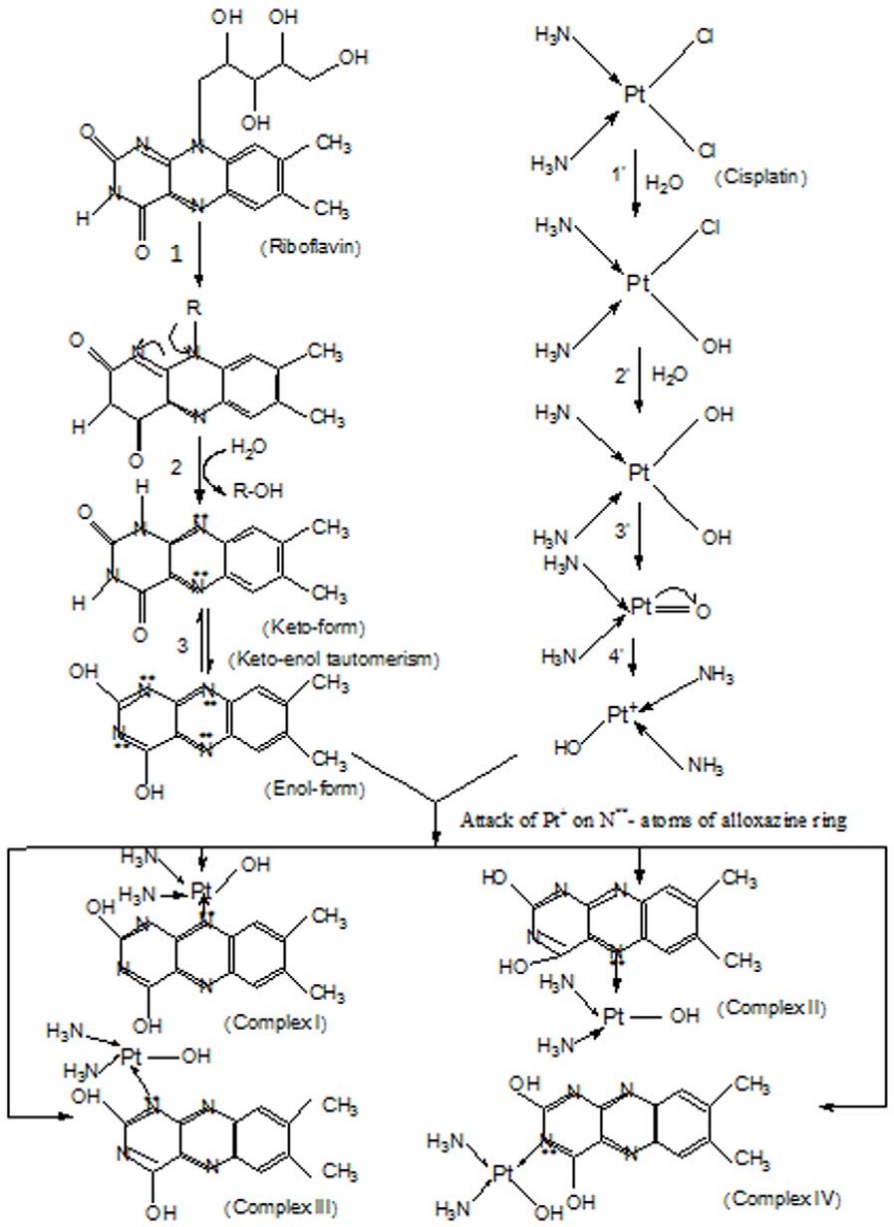
Putative mechanism of complex formation between riboflavin and cisplatin. Bio-activated form of cisplatin may eventually become electron-deficient compound and can interacts with electron-rich alloxazine ring N-atoms of riboflavin (keto-enol forms) in aqueous medium resulting into formation of four complexes of both the compounds.

It is documented that low dose of CP triggers apoptosis in the target cells while necrosis is induced at the higher dose depending upon the alteration of the redox status of the cells [33], [34], [35]. RF and CP individually are known stress inducers in the cells via generation of various free radical(s) that are able to transcend organ(s) damage and cause alteration in biomlecules as well as their respective functions [5], [16] but, their interaction in all probabilities would wane the cellular stress to the level favoring apoptosis as mode of cell death instead of necrosis [35], [36]. This possibly is operative in our case when CP-RF combination is used leading to normalization of all the parameters tested [5]. In addition, RF in the form of FAD/FMN is used in many energy generating pathways including ETC and oxidative phosphorylation. If RF is not sufficient in the cancer patients, the aerobic mode of energy generation shifts to anaerobic glycolysis which is favorable to tumor growth. Hence, supplementation of RF may somehow suppress or at least slow down the tumerogenesis. RF, in the present context, in spite of being proved pro-oxidant, possibly acts as an antioxidant because it has shown quenching of the free radicals generating potential of CP. Hence, in the present study, we propose that the quenching is probably due to molecular interaction between CP and RF via their excitable groups. Although this interaction happens without photoillumination but upon exposure of light, RF being a strong photosensitizer is expected to be excited and hence the process of enolization is favored leading to formation of stronger co-ordination between CP and excited RF. This may be the reason for stronger effect of CP-RF combination under light. Moreover, FTIR analysis vividly indicates that RF undergoes enolization under photoillumination. On the other hand, CP undergoes aquation reaction in water that ultimately forms diammoniumplatinum oxide. The Light exposure can cleave the π- bond between platinum (Pt) and oxygen of diammoniumplatinum oxide in heterolytic fashion, making Pt an electron deficient species (Pt^+^). This highly unstable species can attack at lone pairs of electrons of nitrogen atoms of the alloxazine ring. Thus, Pt^+^ can form four possible complexes through co-ordination bonding thereby engaging most of CP and RF in combination. The RF-CP interaction occurs without exposure to light also because of propensity of RF to interact with CP without being excited. This could be due to the available lone pair of electrons on N-atom in the ring of RF which can co-ordinate with CP easily (Figure 7).

Thus, RF is a potentially strong therapeutic adjuvant for alleviation of the drug induced toxicities and improves its therapeutic potential possibly by assisting in induction of apoptosis as well as autophagy specifically in the cancer cells and the effect becomes stronger in light. Furthermore, the literature suggests that RF can enhance the antineoplastic action of CP and may also increase the sensitivity in CP resistant cancer cells [20], [25], [37], [38], [39]. Hence, supplementation of RF to the cancer patients undergoing CP based chemotherapy will not only help to overcome the CP induced side effects but also increase its anticancer activity in all probabilities. We, therefore suggest with caution prescribing CP-RF combination followed by low dose of radiation as a better treatment strategy for cancer patients as compared to CP alone. However, the anticancer activity of this combination needs to be tested before its clinical application.

## Supporting Information

Figure S1 **Statistical analysis of cells of renal tubular epithelium and liver histopathological sections at normal, apoptotic and necrotic stages.** Statistical analysis of cells per renal tubular epithelium and liver showing normal, apoptotic and necrotic features was done GraphPad Prism 5.
(DOC)

Table S1 **Post hoc analysis of comet tail-length of kidney and liver samples.** Post hoc analysis of comet tail-length of kidney and liver samples of various treatment groups was done by GraphPad Prism 5. Group I: control Group II: RF Group III: CP Group IV: Combination I under photoillumination Group IV′: Combination I without photoillumination Group V: Combination II under photoillumination Group V′: Combination II without photoillumination.
(DOC)
