# Supplementary material for: Riboflavin Ameliorates Cisplatin Induced Toxicities under Photoillumination
Source: PLoS One. 2012 May 2;7(5):e36273. doi: 10.1371/journal.pone.0036273 (PMC3342168; doi:10.1371/journal.pone.0036273)
Supplement: Figure S1 — Statistical analysis of cells of renal tubular epithelium and liver histopathological sections at normal, apoptotic and necrotic stages. Statistical analysis of cells per renal tubular epithelium and liver showing normal, apoptotic and necrotic features was done GraphPad Prism 5. (DOC) [file pone.0036273.s001.doc]

**Statistical Analysis of Renal tubular epithelium cells**

**N**ormal kidney cells

|  | | |  | |  | |  | |  |  |
| --- | --- | --- | --- | --- | --- | --- | --- | --- | --- | --- |
|  | | |  | |  | |  | |  |  |
| **One-way analysis of variance** | | |  | |  | |  | |  |  |
| P value | | | < 0.0001 | |  | |  | |  |  |
| P value summary | | | *** | |  | |  | |  |  |
| Are means signif. different? (P < 0.05) | | | Yes | |  | |  | |  |  |
| Number of groups | | | 4 | |  | |  | |  |  |
| F | | | 26.05 | |  | |  | |  |  |
| R squared | | | 0.8301 | |  | |  | |  |  |
|  | | |  | |  | |  | |  |  |
| Bartlett's test for equal variances | | |  | |  | |  | |  |  |
| Bartlett's statistic (corrected) | | | 3.848 | |  | |  | |  |  |
| P value | | | 0.2783 | |  | |  | |  |  |
| P value summary | | | ns | |  | |  | |  |  |
| Do the variances differ signif. (P < 0.05) | | | No | |  | |  | |  |  |
|  | | |  | |  | |  | |  |  |
| ANOVA Table | | | SS | | df | | MS | |  |  |
| Treatment (between columns) | | | 50.41 | | 3 | | 16.80 | |  |  |
| Residual (within columns) | | | 10.32 | | 16 | | 0.6450 | |  |  |
| Total | | | 60.73 | | 19 | |  | |  |  |
|  | | |  | |  | |  | |  |  |
| **Tukey's Multiple Comparison Test** | | | **Mean Diff.** | | **q** | | **P < 0.05?** | | **Summary** | **95% CI of diff** |
| Control vs RF | | | 2.380 | | 6.626 | | Yes | | ** | 0.9268 to 3.833 |
| Control vs CP | | | 4.340 | | 12.08 | | Yes | | *** | 2.887 to 5.793 |
| Control vs Combination I | | | 1.320 | | 3.675 | | No | | ns | -0.1332 to 2.773 |
| RF vs CP | | | 1.960 | | 5.457 | | Yes | | ** | 0.5068 to 3.413 |
| RF vs Combination I | | | -1.060 | | 2.951 | | No | | ns | -2.513 to 0.3932 |
| CP vs Combination I | | | -3.020 | | 8.408 | | Yes | | *** | -4.473 to -1.567 |
|  | | |  | |  | |  | |  |  |
| Number of values | 5 | 5 | | 5 | | 5 | |  | | |
|  |  |  | |  | |  | |  | | |
| Minimum | 10.30 | 7.500 | | 6.200 | | 8.200 | |  | | |
| 25% Percentile | 10.40 | 7.750 | | 6.250 | | 8.750 | |  | | |
| Median | 10.80 | 8.500 | | 6.600 | | 9.500 | |  | | |
| 75% Percentile | 11.60 | 9.450 | | 7.000 | | 10.60 | |  | | |
| Maximum | 11.60 | 9.700 | | 7.200 | | 11.30 | |  | | |
|  |  |  | |  | |  | |  | | |
| **Mean** | **10.96** | **8.580** | | **6.620** | | **9.640** | |  | | |
| **Std. Deviation** | **0.6107** | **0.8871** | | **0.4025** | | **1.122** | |  | | |
| **Std. Error** | **0.2731** | **0.3967** | | **0.1800** | | **0.5016** | |  | | |
|  |  |  | |  | |  | |  | | |
| Lower 95% CI | 10.20 | 7.478 | | 6.120 | | 8.247 | |  | | |
| Upper 95% CI | 11.72 | 9.682 | | 7.120 | | 11.03 | |  | | |

**Kidney cells under apoptosis**

|  |  |  |  |  |  |
| --- | --- | --- | --- | --- | --- |
|  |  |  |  |  |  |
|  |  |  |  |  |  |
| One-way analysis of variance |  |  |  |  |  |
| P value | 0.0001 |  |  |  |  |
| P value summary | *** |  |  |  |  |
| Are means signif. different? (P < 0.05) | Yes |  |  |  |  |
| Number of groups | 4 |  |  |  |  |
| F | 13.66 |  |  |  |  |
| R squared | 0.7191 |  |  |  |  |
|  |  |  |  |  |  |
| Bartlett's test for equal variances |  |  |  |  |  |
| Bartlett's statistic (corrected) | 26.92 |  |  |  |  |
| P value | < 0.0001 |  |  |  |  |
| P value summary | *** |  |  |  |  |
| Do the variances differ signif. (P < 0.05) | Yes |  |  |  |  |
|  |  |  |  |  |  |
| ANOVA Table | SS | df | MS |  |  |
| Treatment (between columns) | 15.00 | 3 | 4.999 |  |  |
| Residual (within columns) | 5.857 | 16 | 0.3661 |  |  |
| Total | 20.85 | 19 |  |  |  |
|  |  |  |  |  |  |
| **Tukey's Multiple Comparison Test** | **Mean Diff.** | **q** | **P < 0.05?** | **Summary** | **95% CI of diff** |
| Control vs RF | -1.055 | 3.901 | No | ns | -2.150 to 0.03937 |
| Control vs CP | -1.801 | 6.658 | Yes | ** | -2.896 to -0.7066 |
| Control vs Combination I | -2.299 | 8.498 | Yes | *** | -3.394 to -1.205 |
| RF vs CP | -0.7460 | 2.757 | No | ns | -1.841 to 0.3488 |
| RF vs Combination I | -1.244 | 4.598 | Yes | * | -2.339 to -0.1492 |
| CP vs Combination I | -0.4980 | 1.840 | No | ns | -1.593 to 0.5968 |

|  |  |  |  |  |
| --- | --- | --- | --- | --- |
| Number of values | 5 | 5 | 5 | 5 |
|  |  |  |  |  |
| Minimum | 0.0 | 0.8000 | 1.130 | 1.700 |
| 25% Percentile | 0.0060 | 0.8200 | 1.205 | 1.850 |
| Median | 0.0240 | 0.9000 | 1.510 | 2.400 |
| 75% Percentile | 0.0335 | 1.420 | 2.595 | 2.750 |
| Maximum | 0.0380 | 1.880 | 3.610 | 2.900 |
|  |  |  |  |  |
| **Mean** | **0.0206** | **1.076** | **1.822** | **2.320** |
| **Std. Deviation** | **0.01486** | **0.4535** | **1.016** | **0.4764** |
| **Std. Error** | **0.006645** | **0.2028** | **0.4542** | **0.2131** |
|  |  |  |  |  |
| Lower 95% CI | 0.002150 | 0.5129 | 0.5610 | 1.728 |
| Upper 95% CI | 0.03905 | 1.639 | 3.083 | 2.912 |

**Kidney cells under necrosis**

|  |  |  |  |  |  |
| --- | --- | --- | --- | --- | --- |
|  |  |  |  |  |  |
|  |  |  |  |  |  |
| One-way analysis of variance |  |  |  |  |  |
| P value | < 0.0001 |  |  |  |  |
| P value summary | *** |  |  |  |  |
| Are means signif. different? (P < 0.05) | Yes |  |  |  |  |
| Number of groups | 4 |  |  |  |  |
| F | 46.10 |  |  |  |  |
| R squared | 0.8963 |  |  |  |  |
|  |  |  |  |  |  |
| Bartlett's test for equal variances |  |  |  |  |  |
| Bartlett's statistic (corrected) | 65.92 |  |  |  |  |
| P value | < 0.0001 |  |  |  |  |
| P value summary | *** |  |  |  |  |
| Do the variances differ signif. (P < 0.05) | Yes |  |  |  |  |
|  |  |  |  |  |  |
| ANOVA Table | SS | df | MS |  |  |
| Treatment (between columns) | 40.84 | 3 | 13.61 |  |  |
| Residual (within columns) | 4.725 | 16 | 0.2953 |  |  |
| Total | 45.57 | 19 |  |  |  |
|  |  |  |  |  |  |
| **Tukey's Multiple Comparison Test** | **Mean Diff.** | **q** | **P < 0.05?** | **Summary** | **95% CI of diff** |
| Column A vs Column B | -0.0528 | 0.2173 | No | ns | -1.036 to 0.9305 |
| Column A vs Column C | -3.351 | 13.79 | Yes | *** | -4.334 to -2.367 |
| Column A vs Column D | -0.1018 | 0.4189 | No | ns | -1.085 to 0.8815 |
| Column B vs Column C | -3.298 | 13.57 | Yes | *** | -4.281 to -2.314 |
| Column B vs Column D | -0.0490 | 0.2016 | No | ns | -1.032 to 0.9343 |
| Column C vs Column D | 3.249 | 13.37 | Yes | *** | 2.265 to 4.232 |

|  |  |  |  |  |
| --- | --- | --- | --- | --- |
| Number of values | 5 | 5 | 5 | 5 |
|  |  |  |  |  |
| Minimum | 0.0 | 0.0330 | 2.400 | 0.0060 |
| 25% Percentile | 0.0 | 0.0445 | 2.425 | 0.0530 |
| Median | 0.0080 | 0.0610 | 3.280 | 0.1000 |
| 75% Percentile | 0.0145 | 0.0755 | 4.330 | 0.1700 |
| Maximum | 0.0150 | 0.0760 | 5.060 | 0.2000 |
|  |  |  |  |  |
| **Mean** | **0.0074** | **0.0602** | **3.358** | **0.1092** |
| **Std. Deviation** | **0.007266** | **0.01751** | **1.084** | **0.07073** |
| **Std. Error** | **0.003250** | **0.007832** | **0.4850** | **0.03163** |
|  |  |  |  |  |
| Lower 95% CI | -0.001622 | 0.03845 | 2.012 | 0.02137 |
| Upper 95% CI | 0.01642 | 0.08195 | 4.704 | 0.1970 |

**Statistical Analysis of Liver cells**

**No**rmal liver cells

|  |  |  |  |  |  |
| --- | --- | --- | --- | --- | --- |
|  |  |  |  |  |  |
|  |  |  |  |  |  |
| One-way analysis of variance |  |  |  |  |  |
| P value | 0.0009 |  |  |  |  |
| P value summary | *** |  |  |  |  |
| Are means signif. different? (P < 0.05) | Yes |  |  |  |  |
| Number of groups | 4 |  |  |  |  |
| F | 9.176 |  |  |  |  |
| R squared | 0.6324 |  |  |  |  |
|  |  |  |  |  |  |
| Bartlett's test for equal variances |  |  |  |  |  |
| Bartlett's statistic (corrected) | 7.725 |  |  |  |  |
| P value | 0.0521 |  |  |  |  |
| P value summary | ns |  |  |  |  |
| Do the variances differ signif. (P < 0.05) | No |  |  |  |  |
|  |  |  |  |  |  |
| ANOVA Table | SS | df | MS |  |  |
| Treatment (between columns) | 63620 | 3 | 21210 |  |  |
| Residual (within columns) | 36970 | 16 | 2311 |  |  |
| Total | 100600 | 19 |  |  |  |
|  |  |  |  |  |  |
| **Tukey's Multiple Comparison Test** | **Mean Diff.** | **q** | **P < 0.05?** | **Summary** | **95% CI of diff** |
| Control vs RF | 94.60 | 4.400 | Yes | * | 7.617 to 181.6 |
| Control vs CP | 151.8 | 7.061 | Yes | *** | 64.82 to 238.8 |
| Control vs Combination I | 46.20 | 2.149 | No | ns | -40.78 to 133.2 |
| RF vs CP | 57.20 | 2.661 | No | ns | -29.78 to 144.2 |
| RF vs Combination I | -48.40 | 2.251 | No | ns | -135.4 to 38.58 |
| CP vs Combination I | -105.6 | 4.912 | Yes | * | -192.6 to -18.62 |

|  |  |  |  |  |
| --- | --- | --- | --- | --- |
| Number of values | 5 | 5 | 5 | 5 |
|  |  |  |  |  |
| Minimum | 425.0 | 405.0 | 340.0 | 425.0 |
| 25% Percentile | 450.0 | 413.0 | 345.0 | 433.0 |
| Median | 535.0 | 435.0 | 375.0 | 492.0 |
| 75% Percentile | 595.0 | 445.5 | 400.5 | 518.0 |
| Maximum | 625.0 | 450.0 | 416.0 | 531.0 |
|  |  |  |  |  |
| **Mean** | **525.0** | **430.4** | **373.2** | **478.8** |
| **Std. Deviation** | **77.78** | **17.69** | **30.06** | **44.47** |
| **Std. Error** | **34.79** | **7.909** | **13.44** | **19.89** |
|  |  |  |  |  |
| Lower 95% CI | 428.4 | 408.4 | 335.9 | 423.6 |
| Upper 95% CI | 621.6 | 452.4 | 410.5 | 534.0 |

**Liver cells** under apoptosis

|  | | |  | |  | |  | |  |  |
| --- | --- | --- | --- | --- | --- | --- | --- | --- | --- | --- |
|  | | |  | |  | |  | |  |  |
|  | | |  | |  | |  | |  |  |
| **One-way analysis of variance** | | |  | |  | |  | |  |  |
| P value | | | < 0.0001 | |  | |  | |  |  |
| P value summary | | | *** | |  | |  | |  |  |
| Are means signif. different? (P < 0.05) | | | Yes | |  | |  | |  |  |
| Number of groups | | | 4 | |  | |  | |  |  |
| F | | | 115.7 | |  | |  | |  |  |
| R squared | | | 0.9559 | |  | |  | |  |  |
|  | | |  | |  | |  | |  |  |
| Bartlett's test for equal variances | | |  | |  | |  | |  |  |
| Bartlett's statistic (corrected) | | | 8.489 | |  | |  | |  |  |
| P value | | | 0.0369 | |  | |  | |  |  |
| P value summary | | | * | |  | |  | |  |  |
| Do the variances differ signif. (P < 0.05) | | | Yes | |  | |  | |  |  |
|  | | |  | |  | |  | |  |  |
| ANOVA Table | | | SS | | df | | MS | |  |  |
| Treatment (between columns) | | | 30420 | | 3 | | 10140 | |  |  |
| Residual (within columns) | | | 1402 | | 16 | | 87.65 | |  |  |
| Total | | | 31830 | | 19 | |  | |  |  |
|  | | |  | |  | |  | |  |  |
| **Tukey's Multiple Comparison Test** | | | **Mean Diff.** | | **q** | | **P < 0.05?** | | **Summary** | **95% CI of diff** |
| Control vs RF | | | -11.00 | | 2.627 | | No | | ns | -27.94 to 5.940 |
| Control vs CP | | | -7.600 | | 1.815 | | No | | ns | -24.54 to 9.340 |
| Control vs Combination I | | | -95.80 | | 22.88 | | Yes | | *** | -112.7 to -78.86 |
| RF vs CP | | | 3.400 | | 0.8121 | | No | | ns | -13.54 to 20.34 |
| RF vs Combination I | | | -84.80 | | 20.25 | | Yes | | *** | -101.7 to -67.86 |
| CP vs Combination I | | | -88.20 | | 21.07 | | Yes | | *** | -105.1 to -71.26 |
|  |  |  | |  | |  | |  | | |
| Number of values | 5 | 5 | | 5 | | 5 | |  | | |
|  |  |  | |  | |  | |  | | |
| Minimum | 21.00 | 25.00 | | 25.00 | | 106.0 | |  | | |
| 25% Percentile | 21.50 | 28.50 | | 27.00 | | 108.0 | |  | | |
| Median | 25.00 | 35.00 | | 32.00 | | 112.0 | |  | | |
| 75% Percentile | 27.50 | 43.00 | | 37.50 | | 137.0 | |  | | |
| Maximum | 30.00 | 45.00 | | 39.00 | | 142.0 | |  | | |
|  |  |  | |  | |  | |  | | |
| **Mean** | **24.60** | **35.60** | | **32.20** | | **120.4** | |  | | |
| **Std. Deviation** | **3.507** | **7.797** | | **5.541** | | **15.71** | |  | | |
| **Std. Error** | **1.568** | **3.487** | | **2.478** | | **7.026** | |  | | |
|  |  |  | |  | |  | |  | | |
| Lower 95% CI | 20.25 | 25.92 | | 25.32 | | 100.9 | |  | | |
| Upper 95% CI | 28.95 | 45.28 | | 39.08 | | 139.9 | |  | | |

**Liver cells under necrosis**

|  | | |  | |  | |  | |  |  |
| --- | --- | --- | --- | --- | --- | --- | --- | --- | --- | --- |
|  | | |  | |  | |  | |  |  |
|  | | |  | |  | |  | |  |  |
| **One-way analysis of variance** | | |  | |  | |  | |  |  |
| P value | | | < 0.0001 | |  | |  | |  |  |
| P value summary | | | *** | |  | |  | |  |  |
| Are means signif. different? (P < 0.05) | | | Yes | |  | |  | |  |  |
| Number of groups | | | 4 | |  | |  | |  |  |
| F | | | 110.2 | |  | |  | |  |  |
| R squared | | | 0.9538 | |  | |  | |  |  |
|  | | |  | |  | |  | |  |  |
| Bartlett's test for equal variances | | |  | |  | |  | |  |  |
| Bartlett's statistic (corrected) | | | 32.17 | |  | |  | |  |  |
| P value | | | < 0.0001 | |  | |  | |  |  |
| P value summary | | | *** | |  | |  | |  |  |
| Do the variances differ signif. (P < 0.05) | | | Yes | |  | |  | |  |  |
|  | | |  | |  | |  | |  |  |
| ANOVA Table | | | SS | | df | | MS | |  |  |
| Treatment (between columns) | | | 41400 | | 3 | | 13800 | |  |  |
| Residual (within columns) | | | 2003 | | 16 | | 125.2 | |  |  |
| Total | | | 43400 | | 19 | |  | |  |  |
|  | | |  | |  | |  | |  |  |
| **Tukey's Multiple Comparison Test** | | | **Mean Diff.** | | **q** | | **P < 0.05?** | | **Summary** | **95% CI of diff** |
| Column A vs Column B | | | -2.400 | | 0.4796 | | No | | ns | -22.65 to 17.85 |
| Column A vs Column C | | | -107.4 | | 21.46 | | Yes | | *** | -127.6 to -87.15 |
| Column A vs Column D | | | -4.800 | | 0.9592 | | No | | ns | -25.05 to 15.45 |
| Column B vs Column C | | | -105.0 | | 20.98 | | Yes | | *** | -125.2 to -84.75 |
| Column B vs Column D | | | -2.400 | | 0.4796 | | No | | ns | -22.65 to 17.85 |
| Column C vs Column D | | | 102.6 | | 20.50 | | Yes | | *** | 82.35 to 122.8 |
|  |  |  | |  | |  | |  | | |
| Number of values | 5 | 5 | | 5 | | 5 | |  | | |
|  |  |  | |  | |  | |  | | |
| Minimum | 2.000 | 2.000 | | 75.00 | | 5.000 | |  | | |
| 25% Percentile | 2.000 | 3.000 | | 90.50 | | 5.000 | |  | | |
| Median | 3.000 | 5.000 | | 115.0 | | 7.000 | |  | | |
| 75% Percentile | 4.000 | 8.000 | | 128.0 | | 11.00 | |  | | |
| Maximum | 4.000 | 9.000 | | 131.0 | | 12.00 | |  | | |
|  |  |  | |  | |  | |  | | |
| **Mean** | **3.000** | **5.400** | | **110.4** | | **7.800** | |  | | |
| **Std. Deviation** | **1.000** | **2.702** | | **21.97** | | **3.114** | |  | | |
| **Std. Error** | **0.4472** | **1.208** | | **9.826** | | **1.393** | |  | | |
|  |  |  | |  | |  | |  | | |
| Lower 95% CI | 1.758 | 2.045 | | 83.12 | | 3.933 | |  | | |
| Upper 95% CI | 4.242 | 8.755 | | 137.7 | | 11.67 | |  | | |
