# Supplementary material for: Riboflavin Ameliorates Cisplatin Induced Toxicities under Photoillumination
Source: PLoS One. 2012 May 2;7(5):e36273. doi: 10.1371/journal.pone.0036273 (PMC3342168; doi:10.1371/journal.pone.0036273)
Supplement: Table S1 — Post hoc analysis of comet tail-length of kidney and liver samples. Post hoc analysis of comet tail-length of kidney and liver samples of various treatment groups was done by GraphPad Prism 5. Group I: control Group II: RF Group III: CP Group IV: Combination I under photoillumination Group IV′: Combination I without photoillumination Group V: Combination II under photoillumination Group V′: Combination II without photoillumination. (DOC) [file pone.0036273.s002.doc]

| | **Post hoc analysis of kidney cells**  **One-way analysis of variance** |  |  |  |  |  | | --- | --- | --- | --- | --- | --- | | P value | < 0.0001 |  |  |  |  | | P value summary | *** |  |  |  |  | | Are means signif. different? (P < 0.05) | Yes |  |  |  |  | | Number of groups | 7 |  |  |  |  | | F | 3117 |  |  |  |  | | R squared | 0.9730 |  |  |  |  | |  |  |  |  |  |  | | Bartlett's test for equal variances |  |  |  |  |  | | Bartlett's statistic (corrected) | 42.42 |  |  |  |  | | P value | < 0.0001 |  |  |  |  | | P value summary | *** |  |  |  |  | | Do the variances differ signif. (P < 0.05) | Yes |  |  |  |  | |  |  |  |  |  |  | | ANOVA Table | SS | df | MS |  |  | | Treatment (between columns) | 19020 | 6 | 3170 |  |  | | Residual (within columns) | 526.8 | 518 | 1.017 |  |  | | Total | 19550 | 524 |  |  |  | |  |  |  |  |  |  | | **Tukey's Multiple Comparison Test** | **Mean Diff.** | **q** | **< 0.05?** | **Summary** | **95% CI of diff** | | I vs II | -8.704 | 74.74 | Yes | *** | -9.197 to -8.211 | | I vs III | -17.47 | 150.0 | Yes | *** | -17.96 to -16.98 | | I vs IV | -10.84 | 93.12 | Yes | *** | -11.34 to -10.35 | | I vs IV' | -15.84 | 136.1 | Yes | *** | -16.34 to -15.35 | | I vs V' | -3.440 | 29.54 | Yes | *** | -3.933 to -2.947 | | I vs V' | -14.28 | 122.6 | Yes | *** | -14.77 to -13.79 | | II vs III | -8.764 | 75.26 | Yes | *** | -9.257 to -8.271 | | II vs IV | -2.140 | 18.38 | Yes | *** | -2.633 to -1.647 | | II vs IV' | -7.140 | 61.31 | Yes | *** | -7.633 to -6.647 | | II vs V' | 5.264 | 45.20 | Yes | *** | 4.771 to 5.757 | | II vs V' | -5.576 | 47.88 | Yes | *** | -6.069 to -5.083 | | III vs IV | 6.624 | 56.88 | Yes | *** | 6.131 to 7.117 | | III vs IV' | 1.624 | 13.95 | Yes | *** | 1.131 to 2.117 | | III vs V' | 14.03 | 120.5 | Yes | *** | 13.54 to 14.52 | | III vs V' | 3.188 | 27.38 | Yes | *** | 2.695 to 3.681 | | IV vs IV' | -5.000 | 42.94 | Yes | *** | -5.493 to -4.507 | | IV vs V' | 7.404 | 63.58 | Yes | *** | 6.911 to 7.897 | | IV vs V' | -3.436 | 29.51 | Yes | *** | -3.929 to -2.943 | | IV' vs V' | 12.40 | 106.5 | Yes | *** | 11.91 to 12.90 | | IV' vs V' | 1.564 | 13.43 | Yes | *** | 1.071 to 2.057 | | V' vs V' | -10.84 | 93.09 | Yes | *** | -11.33 to -10.35 | |  |  |  |  |  |
| --- | --- | --- | --- | --- | --- | --- | --- | --- | --- | --- | --- | --- | --- | --- | --- | --- | --- | --- | --- | --- | --- | --- | --- | --- | --- | --- | --- | --- | --- | --- | --- | --- | --- | --- | --- | --- | --- | --- | --- | --- | --- | --- | --- | --- | --- | --- | --- | --- | --- | --- | --- | --- | --- | --- | --- | --- | --- | --- | --- | --- | --- | --- | --- | --- | --- | --- | --- | --- | --- | --- | --- | --- | --- | --- | --- | --- | --- | --- | --- | --- | --- | --- | --- | --- | --- | --- | --- | --- | --- | --- | --- | --- | --- | --- | --- | --- | --- | --- | --- | --- | --- | --- | --- | --- | --- | --- | --- | --- | --- | --- | --- | --- | --- | --- | --- | --- | --- | --- | --- | --- | --- | --- | --- | --- | --- | --- | --- | --- | --- | --- | --- | --- | --- | --- | --- | --- | --- | --- | --- | --- | --- | --- | --- | --- | --- | --- | --- | --- | --- | --- | --- | --- | --- | --- | --- | --- | --- | --- | --- | --- | --- | --- | --- | --- | --- | --- | --- | --- | --- | --- | --- | --- | --- | --- | --- | --- | --- | --- | --- | --- | --- | --- | --- | --- | --- | --- | --- | --- | --- | --- | --- | --- | --- | --- | --- | --- | --- | --- | --- | --- | --- | --- | --- | --- | --- | --- | --- | --- | --- | --- | --- | --- | --- | --- | --- | --- | --- | --- | --- | --- | --- | --- | --- | --- | --- | --- | --- | --- | --- | --- | --- | --- | --- | --- | --- | --- | --- | --- | --- | --- | --- | --- | --- | --- | --- | --- | --- | --- | --- | --- | --- |

|  | I | II | III | IV | IV' | V | V' |
| --- | --- | --- | --- | --- | --- | --- | --- |
| Number of values | 75 | 75 | 75 | 75 | 75 | 75 | 75 |
|  |  |  |  |  |  |  |  |
| Minimum | 5.000 | 14.90 | 22.00 | 17.00 | 21.00 | 9.000 | 20.00 |
| 25% Percentile | 7.200 | 15.40 | 24.00 | 18.00 | 23.50 | 10.50 | 21.20 |
| Median | 7.800 | 16.20 | 25.00 | 18.50 | 23.70 | 11.20 | 22.00 |
| 75% Percentile | 8.700 | 17.40 | 26.20 | 19.50 | 24.00 | 11.40 | 22.20 |
| Maximum | 9.000 | 18.00 | 28.00 | 19.80 | 25.00 | 13.00 | 24.00 |
|  |  |  |  |  |  |  |  |
| **Mean** | **7.652** | **16.36** | **25.12** | **18.50** | **23.50** | **11.09** | **21.93** |
| **Std. Deviation** | **0.9852** | **1.068** | **1.457** | **0.8625** | **0.8091** | **0.8566** | **0.8682** |
| **Std. Error** | **0.1138** | **0.1233** | **0.1682** | **0.09959** | **0.09343** | **0.09891** | **0.1003** |
|  |  |  |  |  |  |  |  |
| Lower 95% CI | 7.425 | 16.11 | 24.78 | 18.30 | 23.31 | 10.89 | 21.73 |
| Upper 95% CI | 7.879 | 16.60 | 25.46 | 18.69 | 23.68 | 11.29 | 22.13 |

**Post hoc analysis of liver cells**

|  |  |  |  |  |  |
| --- | --- | --- | --- | --- | --- |
|  |  |  |  |  |  |
|  |  |  |  |  |  |
| **One-way analysis of variance** |  |  |  |  |  |
| P value | < 0.0001 |  |  |  |  |
| P value summary | *** |  |  |  |  |
| Are means signif. different? (P < 0.05) | Yes |  |  |  |  |
| Number of groups | 7 |  |  |  |  |
| F | 2068 |  |  |  |  |
| R squared | 0.9599 |  |  |  |  |
|  |  |  |  |  |  |
| Bartlett's test for equal variances |  |  |  |  |  |
| Bartlett's statistic (corrected) | 24.78 |  |  |  |  |
| P value | 0.0004 |  |  |  |  |
| P value summary | *** |  |  |  |  |
| Do the variances differ signif. (P < 0.05) | Yes |  |  |  |  |
|  |  |  |  |  |  |
| ANOVA Table | SS | df | MS |  |  |
| Treatment (between columns) | 13820 | 6 | 2303 |  |  |
| Residual (within columns) | 576.7 | 518 | 1.113 |  |  |
| Total | 14390 | 524 |  |  |  |
|  |  |  |  |  |  |
| **Tukey's Multiple Comparison Test** | **Mean Diff.** | **q** | **P < 0.05?** | **Summary** | **95% CI of diff** |
| I vs II | -6.468 | 53.09 | Yes | *** | -6.984 to -5.953 |
| I vs III | -14.93 | 122.5 | Yes | *** | -15.44 to -14.41 |
| I vs IV | -8.319 | 68.28 | Yes | *** | -8.834 to -7.803 |
| I vs IV' | -12.71 | 104.3 | Yes | *** | -13.22 to -12.19 |
| I vs V | -1.635 | 13.42 | Yes | *** | -2.150 to -1.119 |
| I vs V' | -10.71 | 87.91 | Yes | *** | -11.23 to -10.20 |
| II vs III | -8.457 | 69.42 | Yes | *** | -8.973 to -7.942 |
| II vs IV | -1.851 | 15.19 | Yes | *** | -2.366 to -1.335 |
| II vs IV' | -6.239 | 51.21 | Yes | *** | -6.754 to -5.723 |
| II vs V | 4.833 | 39.67 | Yes | *** | 4.318 to 5.349 |
| II vs V' | -4.243 | 34.82 | Yes | *** | -4.758 to -3.727 |
| III vs IV | 6.607 | 54.23 | Yes | *** | 6.091 to 7.122 |
| III vs IV' | 2.219 | 18.21 | Yes | *** | 1.703 to 2.734 |
| III vs V | 13.29 | 109.1 | Yes | *** | 12.78 to 13.81 |
| III vs V' | 4.215 | 34.59 | Yes | *** | 3.699 to 4.730 |
| IV vs IV' | -4.388 | 36.02 | Yes | *** | -4.904 to -3.873 |
| IV vs V | 6.684 | 54.86 | Yes | *** | 6.169 to 7.200 |
| IV vs V' | -2.392 | 19.63 | Yes | *** | -2.908 to -1.877 |
| IV' vs V | 11.07 | 90.88 | Yes | *** | 10.56 to 11.59 |
| IV' vs V' | 1.996 | 16.38 | Yes | *** | 1.481 to 2.512 |
| V vs V' | -9.076 | 74.50 | Yes | *** | -9.592 to -8.561 |

|  | I | II | III | IV | IV' | V | V' |
| --- | --- | --- | --- | --- | --- | --- | --- |
| Number of values | 75 | 75 | 75 | 75 | 75 | 75 | 75 |
|  |  |  |  |  |  |  |  |
| Minimum | 5.000 | 11.00 | 20.00 | 13.00 | 17.00 | 7.000 | 15.00 |
| 25% Percentile | 6.000 | 12.00 | 20.40 | 14.00 | 18.00 | 7.500 | 16.20 |
| Median | 6.200 | 13.40 | 21.00 | 15.00 | 19.20 | 8.000 | 17.00 |
| 75% Percentile | 7.200 | 14.00 | 22.00 | 16.00 | 20.50 | 9.000 | 18.00 |
| Maximum | 8.500 | 14.50 | 24.00 | 16.50 | 22.00 | 9.400 | 19.50 |
|  |  |  |  |  |  |  |  |
| **Mean** | **6.583** | **13.05** | **21.51** | **14.90** | **19.29** | **8.217** | **17.29** |
| **Std. Deviation** | **0.8972** | **1.020** | **1.157** | **1.046** | **1.363** | **0.8237** | **0.9899** |
| **Std. Error** | **0.1036** | **0.1178** | **0.1336** | **0.1208** | **0.1573** | **0.09511** | **0.1143** |
|  |  |  |  |  |  |  |  |
| Lower 95% CI | 6.376 | 12.82 | 21.24 | 14.66 | 18.98 | 8.028 | 17.07 |
| Upper 95% CI | 6.789 | 13.29 | 21.77 | 15.14 | 19.60 | 8.407 | 17.52 |
